# A Facile Route to C2-Substituted Imidazolium Ionic Liquids

**DOI:** 10.3390/molecules14062235

**Published:** 2009-06-19

**Authors:** Elliot Ennis, Scott T. Handy

**Affiliations:** Department of Chemistry, Middle Tennessee State University, Murfreesboro, TN 37132, USA

**Keywords:** ionic liquids, *N*-heterocyclic carbenes, alkylation, imidazolium cation, substitution

## Abstract

A convenient route for the preparation of C2-substituted imidazolium ionic liquids is reported. This method involves the alkylation of *N*-heterocyclic carbenes, which are readily generated from the C2-unsubstituted imidazolium ionic liquids. It works well for non-functionalized alkyl chlorides, and less well for alkyl bromides and iodides, likely due to competing elimination reactions. The resulting C2-substituted salts can be transformed into ionic liquids via standard anion metathesis reactions.

## Introduction

Room temperature ionic liquids (RTILs) continue to grow in terms of their range of application and in their general utilization in chemistry and related fields [1,2,3]. Part of the reason for their great utility stems from the variable physical properties that can be accessed by simply changing the anion and cation components of these materials [4,5]. Although many different options are available, the most popular family of RTILs continues to be those based on the imidazolium cation. Within this family, much work has been done to study the influence of the two alkyl groups on nitrogen on the physical properties of these liquids. The C2 position is another source of variability, but much less is known about these materials, likely due to the fact that their preparation requires starting from a new imidazole for each substituent that is to be studied. Although certainly achievable, such a situation does require at least two or three steps for the preparation of each new compound. As such, they are more time and effort intensive to study than the simple C2-unsubstituted compounds.

**Figure 1:**
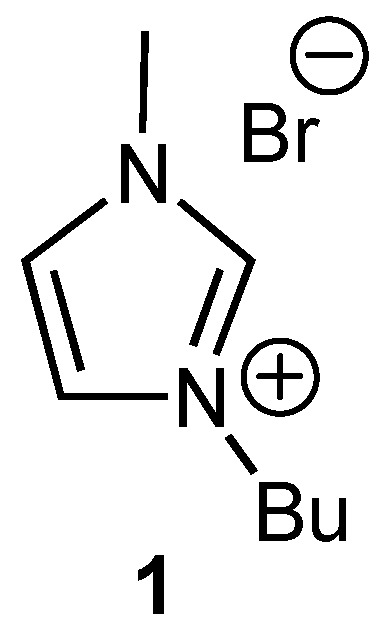
A Simple Imidazolium Ionic Liquid.

The absence of information regarding many C2-substituted imidazolium RTILs is unfortunate, particularly since the unsubstituted salts are incompatible with basic reaction conditions and thus cannot be readily applied as solvents in fundamental reactions such as organometallic additions to carbonyls or the Baylis-Hillman reaction [6,7,8,9]. In an effort to address this limitation and develop a more concise route for the preparation of a variety of C2-substituted imidazolium RTILs, we have considered the possibility of alkylating the *N*-heterocyclic carbenes (NHCs) derived from readily available simple imidazolium RTILs such as **1** (Figure 1).

The attractive features of such an approach are that many RTILs like **1** are commercially available or can be synthesized in a single, simple step. From these materials, a wide range of C2-substituted RTILs could be prepared via standard alkylation chemistry, thereby enabling the preparation of many new RTILs from a single starting material. This would then help to address the limited physical data available regarding such compounds and enable a more accurate prediction regarding the choice of future compounds for application in particular situations.

**Scheme 1:**
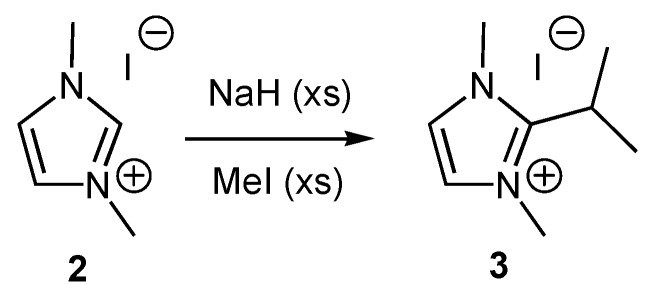
Begtrup Alkylation.

The idea of alkylating NHCs is not completely novel, although it has received very little attention. Begtrup initially reported that the treatment of imidazolium salt **2** with methyl iodide and base (Scheme 1) resulted in alkylation at C2 [10]. Interestingly, this reaction was also able to further alkylate the C2 methyl group by using an excess of methyl iodide and base, ultimately leading to the installation of an isopropyl group at C2 (product **3**). Alder has also reported a few alkylations of simple imidazolium salts by treatment with base and alkyl bromides [11].

## 2. Results and Discussion

**Scheme 2:**
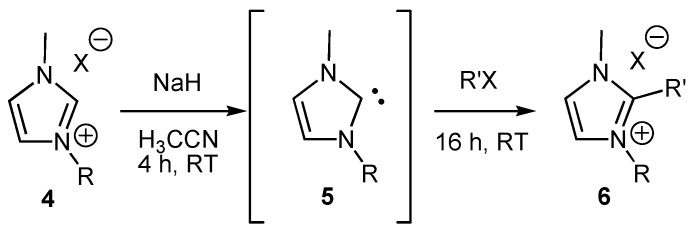
Alkylation Reaction.

To realize this approach to C2-substituted ionic liquids, the *N*-heterocyclic carbene intermediate **5** was first generated from the requisite imidazolium salt **4** using standard conditions (Scheme 2) [12,13]. At this point, the desired alkyl halide was added and the reaction allowed to stir overnight to afford the alkylated product **6**.

Initial attempts employed only slight excesses of base and alkyl halide. These reactions afforded mixtures of starting material and product which proved to be effectively inseparable. However, resubmission of these mixtures to the alkylation conditions did enable eventual complete conversion of all the starting imidazolium salts and never led to further alkylation of the C2 substituent, unlike the reactions of Begtrup. This difference is likely due to the greater steric hindrance imposed by the butyl/methyl combination compared to the dimethyl system studied by Begtrup. The partial conversion in the alkylation reaction is presumably due to competing elimination reactions of the alkyl halides, which is supported by the presence of alkene signals in the ^1^H-NMR of the crude reaction mixtures of reactions with longer alkyl halides (such as chlorodecane).

On the basis of this initial information, further alkylations were conducted using an excess of both the base and the alkyl halide in order to overcome the problems of elimination (Table 1).

The size of the alkyl halide did not appear to have any significant effect on reaction yield as primary alkyl halides ranging in size from two to sixteen carbons were found to be effective reaction partners with the imidazolium carbene intermediate formed in the reaction. On the other hand, the nature of the halide did have a significant impact, with primary alkyl chlorides being superior to their bromo and iodo analogues. Operationally, this can be seen by comparing the number of equivalents of sodium hydride and alkyl halide required for the reaction to proceed to completion. With alkyl chlorides, three equivalents were required to insure complete conversion of the starting imidazolium salt, while 3.5-4 equivalents was required with alkyl bromides and greater than four equivalents with alkyl iodides (Table 1, entries 1, 2, and 3). Again, assuming the elimination reactions are leading to partial conversion, this observation makes sense since elimination reactions are more facile with better leaving groups (bromides and iodides).

Hexadecyl chloride did react successfully. Unfortunately, the alkylated imidazolium RTIL could not be isolated in pure form due to difficulties in removing the excess hexadecyl chloride from the RTIL product. Simple extraction methods, which were successful in all other cases, failed as both the RTIL and hexadecyl chloride were freely miscible with a wide range of solvents including hexanes!

**Scheme 3:**
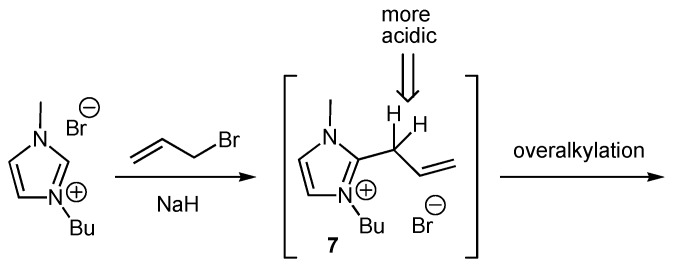
Overalkylation Problems.

This method is not without its limitations. Secondary alkyl halides (such as 2-chloropropane or 2-bromopropane) failed to afford any of the alkylation product. Presumably the increased steric hindrance in these systems results in elimination being the sole reaction pathway. Interestingly, allyl bromide, benzyl bromide, and 4-bromo-1-butene also failed to afford clean alkylated product. In the case of 4-bromo-1-butene, elimination may again be the problem, as starting material was cleanly recovered. For allyl and benzyl bromide, though, the reactions afforded complex mixtures of products, suggesting the alkylation did occur. However, since the initial alkylation product (such as **7**) features even more acidic protons than the starting material, it is quite possible that further alkylation occurs, thereby generating the mixture of products (Scheme 3). No attempts have been made to see if complete alkylation of all acidic protons can be achieved. Finally, 2-bromo ethyl acetate also afforded a complex mixture of products. This is likely due to the same reason that allyl and benzyl bromide failed – overalkylation. As a result, functionalized alkyl groups appear to be problematic substrates for this reaction.

**Scheme 4:**
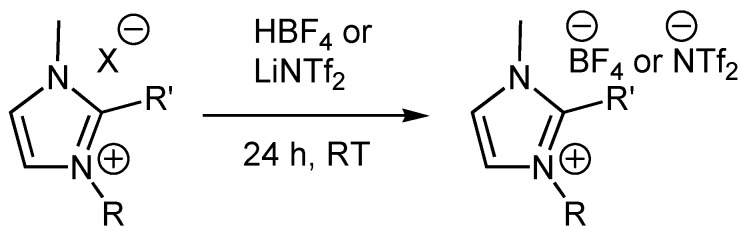
Anion Metathesis.

Imidazolium halides are typically not room temperature liquids, so it was not surprising that only one of the alkylation products, BBMIM, was a liquid. Interestingly, though, all of the remaining halides were relatively low melting solids [14]. Still, to demonstrate that the alkylation route could serve to access more conventional materials, a series of tetrafluoroborate and triflimide salts were prepared via anion metathesis reactions. For these reactions (Scheme 4), the appropriate imidazolium halide was added to an aqueous solution of either fluoroboric acid or lithium bis(trifluoromethanesulfonyl)imide and allowed to stir overnight [15,16].

In general, the tetrafluoroborate RTILs were obtained in modest yields, while the triflimides were obtained in good yields (Table 2). The low yield of the tetrafluoroborate reactions is probably due to the product being at least partially soluble in water (see experimental for full details). As a result, washing them to remove excess fluoroboric acid certainly resulted in at least some loss of material. It is probable that better yields can be obtained via other metathesis techniques [17,18].

In terms of the physical properties, viscosities and melting points were measured for each compound and are reported in Table 3 [19]. Of the trisubstituted butyl-methyl imidazolium halides, the butyl substituted compound was the only one that was a liquid at room temperature, the others were all solids with melting points around 50 °C. These salts became liquid when the halogen group was replaced with a tetrafluoroborate group. All BF_4_ compounds were liquids at room temperature with viscosities ranging from 400-1,170 cP. For BBMIM, a triflimide salt was synthesized and its viscosity compared to the other BBMIM compounds (Table 3, entries 2, 3, and 4). As expected, its viscosity (224 cP) was less than both the tetrafluoroborate (400 cP) and bromide (1,760 cP) analogs [20]. For the sake of comparison, 2-butyl-1-ethyl-3-methyl imidazolium iodide (EBMIM I), tetrafluoroborate, and triflimide RTILs were synthesized and the properties measured. It was found that the EBMIM I was a solid with a melting point of 47-55° C. The tetrafluoroborate analog was less viscous (220 cP) than the iodide and the triflimide was even less viscous (48.3 cP). This trend is consistent with the trend observed for the BBMIM RTILs. The regioisomer 1-butyl-2-ethyl-3-methyl imidazolium bromide (BEMIM Br) was synthesized to allow for the comparison of properties with EBMIM I. Like EBMIM I, BEMIM Br was a solid with a similar melting point (46-52° C). It may be beneficial to perform metathesis reaction on BEMIM Br to form its tetrafluoroborate and triflimide salts, and to compare their physical properties to their corresponding EBMIM analogs.

## Experimental

### General

NMR spectra were collected as solutions in deuterochloroform on either a JEOL 500 or 300 spectrometer. IR spectra were collected on a Varian 800 FTIR as thin films or, for solid samples, neat using an ATR attachment. All solvents and reagents were used as received and all reactions were run in oven-dried glassware under an atmosphere of argon. TLCs were performed on Merck aluminum-backed plates coated with silica and visualized using a UV lamp. Viscosities were measured on a Brookfield DV-E viscometer using materials that had been dried overnight under vacuum at 100 °C. Melting points were measured on a Fisher-Johns hot stage and are uncorrected.

### General procedure for the alkylation of 1-butyl-3-methylimidazolium bromide (BMIM Br) with alkyl chlorides

To a stirred solution of BMIM Br (10 g, 45.6 mmol) in acetonitrile (175 mL) was added NaH (60% in mineral oil) (2.18 g, 54.8 mmol). After allowing the mixture to stir for 4 hours, chlorobutane (16.93 g, 182.9 mmol) was added and the reaction was stirred overnight. The solution was filtered to remove any precipitated NaCl and the resulting solution was then evaporated to dryness to afford a red/orange oil. The resulting oil was washed with ether (3 x 75 mL) to remove any excess alkyl halide and the residual volatiles were then removed under vacuum.

### Synthesis of 1-butyl-2-ethyl-3-methylimidazolium bromide (BEMIM Br)

To a stirred solution of BMIM Br (1.00 g, 4.56 mmol) in acetonitrile (25 mL) was added NaH (60% in mineral oil) (0.22 g, 5.48 mmol). After allowing the mixture to stir for 4 hours, ethyl bromide (2.00 g, 18.3 mmol) was added and the reaction stirred overnight. The solution was filtered to remove any precipitated NaBr and the resulting solution was then evaporated to dryness to afford a red/orange oil. The resulting oil was washed with ether (3 x 20 mL) to remove any excess alkyl halide and the residual volatiles were then removed under vacuum.

### Synthesis of 2-butyl-1-ethyl-3-methylimidazolium iodide (EBMIM I)

To a stirred solution of 1-ethyl-3-methylimidazolium iodide (EMIM I) (2.00 g, 8.37 mmol) in acetonitrile (50 mL) was added NaH (60% in mineral oil) (0.42 g, 10.88 mmol). After allowing the mixture to stir for 4 hours, chlorobutane (2.33 g, 25.1 mmol) was added and the reaction was allowed to stir overnight. The solution was filtered to remove any precipitated NaCl and the resulting solution was then evaporated to dryness to afford a red/orange oil. The resulting oil was washed with ether (3 x 20 mL) to remove any excess alkyl halide and the residual volatiles were then evaporated under vacuum.

### Representative Anion Metathesis to afford Tetrafluoroborate Salts

To a solution of BBMIM-Br (1.55 g, 5.63 mmol) in water (40 mL) was added dropwise HBF_4_ (50 wt% solution) (0.742 g, 8.44 mmol). The mixture was then allowed to stir overnight. The resulting solution was extracted with dichloromethane (3 x 20 mL). The organic layer was collected and then concentrated *in vacuo* and the crude ionic liquid was washed with water until the pH of the extracts was between 6 and 7. The solution was then dried with Na_2_SO_4_. Any residual volatiles were then removed under vacuum. 

### Representative Anion Metathesis to afford Triflimide Salts

To a solution of BBMIM-Br (12.24 g, 44.4 mmol) in water (100 mL) was added lithium bis(trifluoromethanesulfonyl)imide (LiNTf_2_) (20.38 g, 71 mmol). The mixture was then allowed to stir overnight. The resulting solution was extracted with dichloromethane (3 x 75 mL) and the extracts were combined and dried with Na_2_SO_4_. The dried organic layer was then concentrated *in vacuo.*

*1-Butyl-2-ethyl-3-methylimidazolium bromide*: ^1^H-NMR (300 MHz) δ = 7.71 (d, 1H, *J* = 2.07 Hz), 7.52 (d, 1H, *J* = 2.07 Hz), 4.11 (t, 2H), 4.09 (s, 3H), 3.07 (q, 2H), 1.97-1.74 (m, 2H), 1.54-1.00 (m, 5H), 0.82-0.62 (m, 3H); ^13^C-NMR (75 MHz): δ = 147.27, 123.36, 121.32, 48.42, 35.86, 32.26, 19.54, 17.39, 13.54, 11.69; IR (neat): 3,124, 2,961, 2,928, 2,872, 1,637, 1,530, 1,192, 1,033 cm^-1^; HRMS (EI) calcd. for C_10_H_19_N_2_ 167.1548, found 167.1550.

*1, 2-Dibutyl-3-methylimidazolium bromide*: ^1^H-NMR (300 MHz) δ = 7.79 (d, 1H, *J* = 2.07 Hz), 7.52 (d, 1H, *J* = 2.04 Hz), 4.11 (t, 2H), 3.99 (s, 3H), 3.02 (t, 2H), 1.81-1.62 (m, 2H), 1.58-1.43 (m, 2H), 1.41-1.22 (m, 4H), 0.88-0.82 (m, 6H); ^13^C-NMR (75 MHz) δ = 146.49, 123.56, 121.35, 48.46, 36.02, 32.24, 29.29, 23.60, 22.42, 19.68, 13.62, 13.58; IR (neat): 3,148, 2,924, 2,857, 1,688, 1,530, 1,190, 1,033 cm^-1^; HRMS (EI) calcd. for C_12_H_23_N_2_ 195.1861, found 195.1860.

*1-Butyl-2-hexyl-3-methylimidazolium bromide*: ^1^H-NMR (300 MHz) δ = 7.84 (d, 1H, *J* = 2.07 Hz), 7.59 (d, 1H, *J* = 2.07 Hz), 4.14 (t, 2H), 3.99 (s, 3H), 3.04 (t, 2H), 1.86-1.73 (m, 2H), 1.64-1.51 (m, 2H), 1.45-1.18 (m, 8H) 0.96-0.78 (m, 6H); ^13^C-NMR (75 MHz) δ = 146.50, 123.70, 121.34, 48.52, 36.06, 32.29, 31.24, 28.95, 27.33, 23.98, 22.42, 19.74, 13.99, 13.63; IR (neat): 3,124, 2,935, 2,959, 2,873, 1,665, 1,530, 1,466, 1,033 cm^-1^; HRMS (EI) calcd. for C_14_H_27_N_2_ 223.2174, found 223.2171.

*1-Butyl-2-heptyl-3-methylimidazolium bromide*: ^1^H-NMR (300 MHz) δ = 7.74 (d, 1H, *J* = 2.07 Hz), 7.54 (d, 1H, *J* = 2.07 Hz), 4.06 (t, 2H), 3.89 (s, 3H), 2.96 (t, 2H), 1.75-1.61 (m, 2H), 1.56-1.44 (m, 2H), 1.33-1.07 (m, 10H) 0.82 (t, 3H), 0.72 (t, 3H); ^13^C-NMR (75 MHz) δ = 146.38, 123.48, 121.33, 48.38, 35.92, 32.19, 31.38, 29.06, 28.65, 27.22, 23.74, 22.41, 19.56, 13.94, 13.51; IR (neat): 3,054, 2,958, 2,934, 2,820, 1,672, 1,530, 1,465, 1,033 cm^-1^; HRMS (EI) calcd. for C_15_H_29_N_2_ 237.2331, found 237.2333.

*1-Butyl-2-decyl-3-methylimidazolium bromide*: ^1^H-NMR (300 MHz) δ = 7.65 (d, 1H *J* = 2.07 Hz), 7.47 (d, 1H, *J* = 2.07 Hz), 4.00 (t, 2H), 3.82 (s, 3H), 3.33, (t, 2H), 1.72-1.52 (m, 2H), 1.49-1.37 (m, 2H), 1.29-0.98 (m, 16H), 0.78 (t, 3H), 0.68 (t, 3H); ^13^C-NMR (75 MHz) δ = 146.55, 123.74, 121.33, 48.53, 36.06, 32.29, 31.87, 29.51, 29.39, 29.29, 29.15, 27.38, 24.02, 22.7, 19.76, 14.16, 13.64; IR (neat): 3,148, 2,925, 2,855, 1,672, 1,531, 1,466, 1,033 cm^-1^; HRMS (EI) calcd. for C_18_H_35_N_2_ 279.2800, found 279.2801.

*1, 2-Dibutyl-3-methylimidazolium tetrafluoroborate*: ^1^H-NMR (500 MHz) δ = 7.29 (d, 1H, *J* = 2.04 Hz), 7.26 (d, 1H, *J* = 2.07 Hz), 3.99 (t, 2H), 3.75 (s, 3H), 2.90 (t, 2H), 1.84-1.76 (m, 2H) 1.64-1.57 (m, 2H), 1.49-1.34 (m, 4H), 0.92-0.81 (m, 6H); ^13^C-NMR (125 MHz) δ = 146.40, 122.77, 120.76, 47.87, 34.77, 31.76, 28.76, 22.51, 22.05, 19.25, 13.25, 13.23; ^19^F-NMR (470 MHz) δ = -150.98; IR: 3,127, 2,966, 2,937, 2,866, 1,531, 1,466, 1,033 cm^-1^; HRMS (EI) calcd. for C_12_H_23_N_2_ 195.1861, found 195.1861.

*1-Butyl-2-hexyl-3-methylimidazolium tetrafluoroborate*: ^1^H-NMR (500 MHz) δ = 7.67 (d, 1H, *J* = 2.07 Hz), 7.49 (d, 1H, *J* = 2.04 Hz), 4.05 (t, 2H), 3.87 (s, 3H), 2.92 (t, 2H), 1.76-1.64 (m, 2H), 1.56-1.44 (m, 2H), 1.35-1.11 (m, 8H) 0.87-0.70 (m, 6H); ^13^C-NMR (125 MHz) δ=146.55, 122.98, 120.97, 48.05, 34.91, 31.94, 31.11, 28.74, 26.91, 22.94, 22.33, 19.47, 13.88, 13.39; ^19^F-NMR (470 MHz_3_) δ = -150.98; IR: 3,144, 2,966, 2,936, 2,866, 1,532, 1,469, 1,053 cm^-1^; HRMS (EI) calcd. for C_14_H_27_N_2_ 223.2174, found 223.2173.

*1-Butyl-2-heptyl-3-methylimidazolium tetrafluoroborate*: ^1^H-NMR (500 MHz) δ=7.39 (d, 1H, *J* = 2.3 Hz), 7.36 (d, 1H, *J* = 2.3 Hz), 4.08 (t, 2H), 3.85 (s, 3H), 2.99 (t, 2H), 1.72-1.63 (m, 2H), 1.54-1.44 (m, 2H), 1.34-1.06 (m, 10H), 0.83 (t, 3H), 0.75 (t.3H); ^13^C-NMR (125 MHz) δ = 146.26, 122.65, 120.68, 47.76, 34.66, 31.65, 31.11, 28.70, 28.32, 26.70, 22.64, 22.13, 19.14, 13.65, 13.11; ^19^F-NMR (470 MHz) δ = -151.98; IR: 3,220, 2,983, 2,940, 2,826, 1,507, 1,318, 1,022 cm^-1^; RMS (EI) calcd. for C_15_H_29_N_2_ 237.2331, found 237.2333.

*1-Butyl-2-decyl-3-methylimidazolium tetrafluoroborate*: ^1^H-NMR (500 MHz) δ = 7.38 (d, 1H, *J* = 2.3 Hz), 7.35 (d, 1H, *J* = 2.3 Hz), 4.06 (t, 2H), 3.81 (s, 3H), 2.97 (t, 2H) 1.84-1.73 (m, 2H), 1.64-1.56 (m, 2H), 1.44-1.20 (m, 16H), 0.96 (t, 3H), 0.87 (t, 3H); ^13^C-NMR (125 MHz) δ = 146.37, 122.82, 120.78, 47.91, 34.81, 31.78, 31.59, 29.24, 29.11, 29.01, 28.94, 28.83, 26.86, 22.82, 22.41, 19.31, 13.86, 13.25; ^19^F-NMR (470 MHz) δ = -152.01; IR (neat) 3,144, 2,960, 2,924, 2,854, 1,532, 1,466, 1,052 cm^-1^; HRMS (EI) calcd. for C_18_H_35_N_2_ 279.2800, found 279.2802.

*2-Butyl-1-ethyl-3-methylimidazolium iodide*: ^1^H-NMR (500 MHz) δ = 7.32 (d, 1H, *J* = 2.07 Hz), 7.29 (d, 1H, *J* = 2.07 Hz), 3.94 (q, 2H), 3.63 (s, 3H), 2.76 (t, 2H), 1.34-1.06 (m, 7H), 0.60 (t, 3H); ^13^C- NMR (125 MHz) δ = 146.45, 123.24, 120.75, 43.91, 36.31, 29.21. 23.84, 22.31, 15.23, 13.42; IR (neat): 3.095, 2.966, 2.964, 2.867, 1.637, 1.597, 1.397, 1010 cm^-1^; HRMS (EI) calcd. for C_10_H_19_N_2_ 167.1548, found 167.1549.

*2-Butyl-1-ethyl-3-methylimidazolium tetrafluoroborate*: ^1^H-NMR (500 MHz) δ = 7.18 (d, 1H, *J* = 2.3 Hz), 7.17 (d, 1H, *J* = 1.7 Hz), 3.96 (q, 2H), 3.63 (s, 3H), 2.79 (t, 2H), 1.40 (m, 2H), 1.32-1.19 (m, 5H), 0.74 (t, 3H); ^13^C-NMR (125 MHz) δ = 145.96, 122.42, 119.90, 42.86, 34.43, 28.35, 22.16, 21.65, 14.74, 12.88; ^19^F-NMR (470 MHz) δ = -151.91; IR (neat): 3,148, 2,966, 2,941, 2,876, 1,727, 1,532, 1,361, 1,056 cm^-1^; HRMS (EI) calcd. for C_10_H_19_N_2_ 167.1548, found 167.1546.

*2-Butyl-1-ethyl-3-methylimidazolium triflimide*: ^1^H-NMR (500 MHz) δ = 7.28 (d, 1H, *J* = 2.3 Hz), 7.25 (d, 1H, *J* = 2.3 Hz), 4.13 (q, 2H), 3.81 (s, 3H), 2.96 (t, 2H), 1.65-1.58 (m, 2H), 1.53-1.40 (m 5H), 0.97, (t, 3H); ^13^C-NMR (125 MHz) δ = 192.51, 112.71, 120.09, 119.28 (q, *J* = 316 Hz, 2C), 43.33, 34.86, 28.66, 22.71, 22.05, 14.82, 13.09; ^19^F-NMR (470 MHz) δ= -79.03; IR (neat): 3,148, 2,972, 2,943, 2,876, 1,712, 1,532, 1,350, 1,183, 1,055 cm^-1^; HRMS (EI) calcd. for C_10_H_19_N_2_ 167.1548, found 167.1546.

*1, 2-Dibutyl-3-methylimidazolium triflimide*: ^1^H-NMR (500 MHz) δ = 7.19 (app s, 2H), 4.00 (t, 2H), 3.77 (s, 3H), 2.90 (t, 2H), 1.81-1.72 (m, 2H), 1.60-1.52 (m, 2H), 1.45-1.14 (m, 4H), 0.96-0.77 (m, 6H); ^13^C-NMR (125 MHz) δ = 146.26, 122.69, 120.69, 119.61 (q, *J* = 320 Hz, 2C), 53.38, 48.09, 34.99, 31.64, 28.72, 22.78, 22.11, 19.28, 13.19;^19^F-NMR (470 MHz) δ = -78.97; IR (neat): 3,143, 2,937, 2,922, 2,876, 1,667, 1,464, 1,531, 1,349, 1,135, 1,054 cm^-1^; HRMS (EI) calcd. for C_12_H_23_N_2_ 195.1861, found 195.1860.

## Conclusions

Because of the acidic C2 proton, imidazolium ionic liquids are limited to reactions that do not employ strongly basic conditions. The present study set out to investigate if it was possible to devise a method to add groups other than methyl to the C2 position of imidazolium RTILs and to investigate what effect it would have on the physical properties of these molten salts. Additionally, salts with other anions were prepared to determine if the anion effects would be similar to that observed with unsubstituted imidazolium RTILs. Future work is directed at preparing further triflimide salts as they were easy to synthesize and isolate and are less viscous than either the halide or tetrafluoroborate RTILs. Other work will explore a broader range of functionalized alkyl halides, particularly ones with the functional group further removed from the alkyl halide than in the cases explored in the present work.

## Figures and Tables

**Table 1:** Alkylation Results.

Entry	Alkyl Halide	Equivalents	% yield^a^
1	Iodoethane	5	99
2	Bromoethane	4	99
3	Chlorobutane	3	92
4	Iodobutane^a^	3	(50)
5	Chlorohexane	3	85
6	Bromohexane^a^	3	(93)
7	Chloroheptane	3	67
8	Bromoheptane^a^	3	(70)
9	Chlorodecane	3	85
10	Chlorohexadecane	3	NA^b^
11	Bromobutane^a^	3	(96)

^a^ % conversion is shown in paranthesis for reactions that did not proceed to completion; ^b^ precise yield could not be determined due to difficulties in removing the excess alkyl halide and alkene by-product.

**Table 2:** Metathesis Reaction Results.

Entry	R	R’	Anion	% Yield
1	Bu	Bu	BF_4_	53
2	Bu	Hex	BF_4_	64
3	Bu	Heptyl	BF_4_	54
4	Bu	Decyl	BF_4_	56
5	Bu	Bu	NTf_2_	71
6	Et	Bu	BF_4_	44
7	Et	Bu	NTf_2_	83

**Table 3:** Physical Properties of ionic liquids.

Entry	R	R’	Anion	MP	Visc.
1	Bu	Et	Br	46-52	NA
2	Bu	Bu	Br	L	1,760
3	Bu	Bu	BF_4_	L	400
4	Bu	Bu	NTf_2_	L	224
5	Bu	Hex	Br	20ish	NA
6	Bu	Hex	BF_4_	L	1,170
7	Bu	Heptyl	Br	48-55	NA
8	Bu	Heptyl	BF_4_	L	963
9	Bu	Decyl	Br	50-60	NA
10	Bu	Decyl	BF_4_	L	380
11	Et	Bu	I	47-55	NA
12	Et	Bu	BF_4_	L	220
13	Et	Bu	NTf_2_	L	48.3

L = liquid at room temperature. NA = not applicable.
